# Effects of exosomes on pre-metastatic niche formation in tumors

**DOI:** 10.1186/s12943-019-0995-1

**Published:** 2019-03-11

**Authors:** Yaxin Guo, Xiang Ji, Jinbo Liu, Dandan Fan, Quanbo Zhou, Chen Chen, Weiwei Wang, Guixian Wang, Haijiang Wang, Weitang Yuan, Zhenyu Ji, Zhenqiang Sun

**Affiliations:** 1 Department of Colorectal Surgery, The First Affiliated Hospital of Zhengzhou University, Zhengzhou, 450052 Henan China; 2 Henan Academy of Medical and Pharmaceutical Sciences, Zhengzhou University, Zhengzhou, 450052 Henan China; 3 Academy of Medical Sciences, Zhengzhou University, Zhengzhou, 450052 Henan China; 4 School of Basic Medical Sciences, Zhengzhou University, Zhengzhou, 450001 Henan China; 5 Department of Gastrointestinal Surgery, Affiliated Tumor Hospital, Xinjiang Medical University, Ürümqi, 830011 Xinjiang China; 6 Department of Pathology, The First Affiliated Hospital, Zhengzhou University, Zhengzhou, 450052 Henan China

**Keywords:** Exosomes, Pre-metastatic niche, Tumor microenvironment, Immunoregulation, Biomarker

## Abstract

A pre-metastatic niche is a microenvironment prepared for the colonization of circulating tumor cells in specific organs. Exosomes are extracellular vesicles with a variety of biological functions. Exosomes play an irreplaceable role in the development of pre-metastatic niches, and mainly function as communication medium. In this review, we analyzed the effects of exosomes on pre-metastatic niches from various perspectives, including inflammation, immune response, angiogenesis, organotropism, matrix remodeling and biomarker expression. In particular, exosomes express programmed death ligand 1 (PD-L1) and cause the immune escape of tumor cells. The immunomodulatory effects of exosomes and their potential in liquid diagnosis have drawn our attention. The potential value of exosomes and pre-metastatic niches will be realized in the field of immunity therapy.

## Background

Tumor metastasis usually indicates a poor prognosis and is the leading cause of cancer-related death [1]. Thus, meaningful research is being done on how to prevent tumor metastasis. In recent years, the concept of a pre-metastatic niche has provided new ideas to prevent tumor metastasis. The pre-metastatic niche refers to the microenvironment, which is well prepared for tumor cells to colonize in and disseminate to distant organ sites. Lyden firstly proposed the idea of the pre-metastatic niche [2]. The significance of the pre-metastatic niche has received increasing attention in recent years [3, 4]. Cao summarized the characteristics and four stages of the pre-metastatic niche. The key components of the pre-metastatic niche include tumor-derived secreted factors (TDSFs), extracellular vesicles (EVs), bone marrow-derived cells (BMDCs), suppressive immune cells and host stromal cells [5].

Exosomes are extracellular vesicles with a diameter of 30–150 nm [6–8]. Exosomes were found in blood, urine, saliva and other bodily fluids [9]. As a key player in intercellular communication, exosomes transmit information through their cargo levels, including proteins, lipids, DNA, messenger RNA and microRNAs [10–15]. Exosome-mediated intercellular communication mainly occurs in the following three ways: First, the exosome membrane protein can bind to the target cell membrane protein, thereby activating the signal pathway in the target cell. Second, in the extracellular matrix, a protease cleaves the exosome membrane protein, which then binds to receptors on the cell membrane to activate the signaling pathway within the cell. Third, the exosome membrane can directly fuse with the target cell membrane, causing nonselective release of the proteins, mRNA and microRNA of the exosomes [16, 17]. Recently, reports have shown that the formation of a pre-metastatic niche depends on tumor-derived exosomes [2, 12, 18–20]. The function of exosomes depends on the type of cells from which they are derived [21, 22]. Studies have shown that tumor-derived exosomes are involved in the exchange of genetic information between tumor cells and basal cells, resulting in the production of a large number of new blood vessels, which promotes tumor growth and invasion [23, 24].

This article summarizes the role of exosomes in the pre-metastasis niche, to identify new means of cancer immunotherapy. Screening for biomarkers contained within exosomes can provide diagnostic and prognostic value.

## Exosomes promote the upregulation of inflammatory molecules in the pre-metastatic niche

Chronic inflammation is a driving force for tumor development and metastasis. Thus, the local inflammatory microenvironment is one of the basic factors for the formation of a pre-metastatic niche.

Hoshino reported the regulatory effect of integrins (ITGs) on the proinflammatory factor S100. They identified differentially expressed genes (DEGs) by RNA sequencing in Kupffer cells (KCs) treated with different cell-derived exosomes. The results showed that S100A8 and S100P were upregulated more than four-fold after treatment with BxPC-3-LiT exosomes compared to exosomes produced by BxPC-3-LiT ITGβ5KD (BxPC-3-LiT cells with knocked down ITGβ5 expression). After treatment of WI-38 fibroblasts with 4175-LuT exosomes, S100A4, S100A6, S100A10, S100A11, S100A13 and S100A16 were upregulated five-fold compared to the 4175-LuT ITGβ4KD (4175-LuT cells with knocked down ITGβ4 expression) exosomes. Thus, exosome integrins could upregulate the expression of proinflammatory S100 molecules in the distant tissue microenvironment. How do tumor-derived exosomes upregulate the expression of proinflammatory S100? The activation of Src, and its subsequent phosphorylation, may be the main pathway [25].

The local inflammatory microenvironment can induce tumor cells to produce tumor-derived secreted factors (TDSFs), such as vascular endothelial growth factor (VEGF), tumor necrosis factor alpha (TNF-α), transforming growth factor-β (TGF-β) and interleukin (IL). These TDSFs in turn affect myeloid cells through paracrine means to stimulate their migration to future pre-metastatic niche formation sites [26]. Under the stimulation of TDSFs, host stromal cells in the pre-metastatic niche may upregulate the expression of inflammatory factors. BMDCs or immune cells are recruited to the pre-metastatic niche and accelerate the secretion of inflammatory factors. In addition, tumor-derived exosomes carry inflammatory factors into the bloodstream, which in turn reach the pre-metastatic niche. Through the above possible ways, an inflammatory microenvironment favorable for tumors is finally formed in the pre-metastatic niche.

## Exosomes induce immune suppression or immune surveillance in the pre-metastatic niche

### Immune escape induced by exosome-derived PD-L1

In recent years, programmed death receptor 1 (PD-1) and programmed death ligand 1 (PD-L1) have attracted much attention. PD-1 is mainly expressed on macrophages, activated T cells and B cells, while PD-L1 is highly expressed in tumor tissues, tumor-associated antigen-presenting cells (APCs) and stromal cells [27–30]. Under normal circumstances, T cells can recognize and attack tumor cells. However, when PD-1 binds to PD-L1, it provides an inhibitory signal, induces T cell apoptosis and inhibits T cell activation and proliferation. Therefore, blocking the PD-1/PD-L1 pathway can enhance the killing effect of T cells and improve the immune response [31, 32].

Currently, immunotherapy with PD-1/PD-L1 inhibitors has moved from the laboratory to clinical applications. Anti-PD-1/PD-L1 antibodies have fewer adverse effects than conventional radiotherapy and chemotherapy. More importantly, the patient’s remission period was longer after immunotherapy. Anti-PD-1 antibodies have shown reliable effects in clinical treatment [33]. However, patients treated with PD-1/PD-L1 inhibitors have significant individual differences [34, 35]. Studies have shown that patients with high expression of PD-L1 have better clinical efficacy with PD-1/PD-L1 blockers [36]. Researchers demonstrated that histone deacetylase (HDAC) inhibitors combined with anti-PD-1 immunotherapy enhanced the therapeutic efficiency in a melanoma mouse model [37]. Therefore, the direction of future research is how to improve the remission rate, expand the indications for anti-PD-1/PD-L1 antibodies, and reduce adverse reactions.

**Fig. 1:**
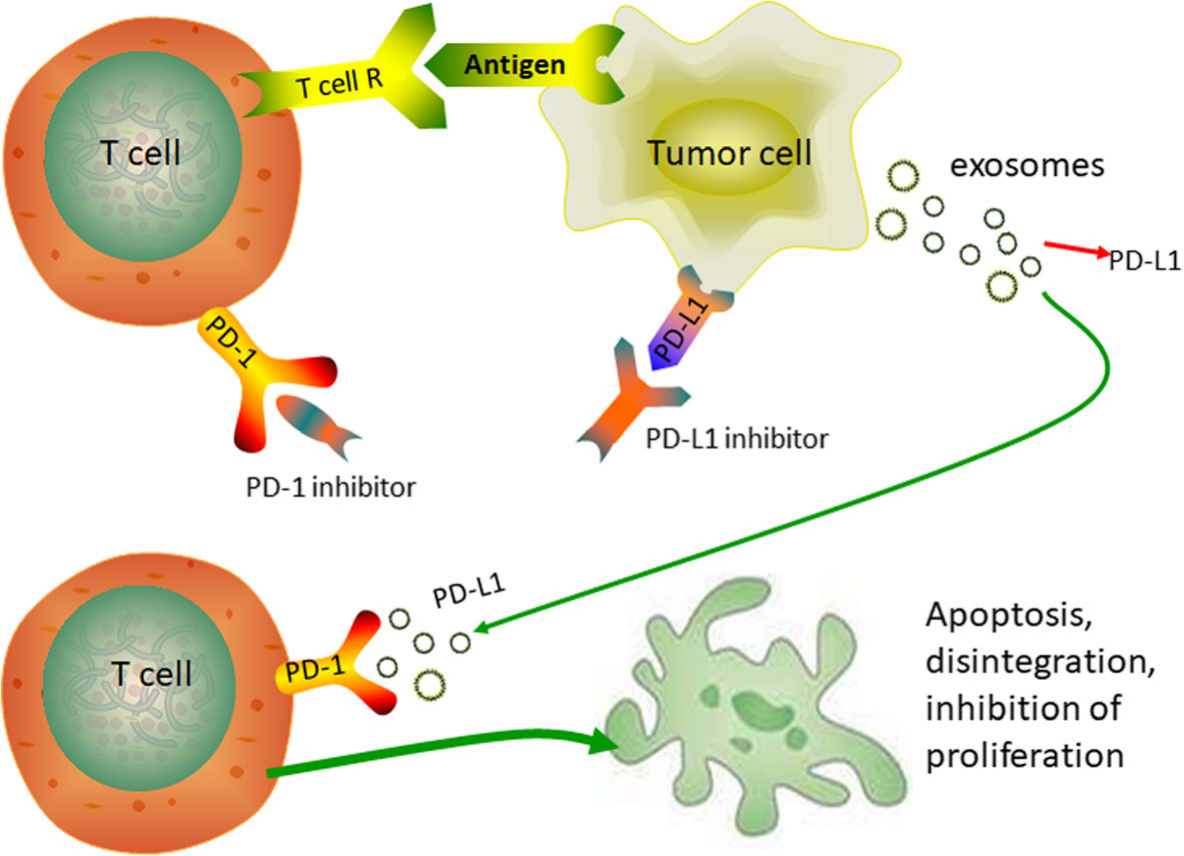
Exosome PD-L1 has a similar function to tumor PD-L1. Exosome PD-L1 can also bind to PD-1 on T cells, induce T cell apoptosis, and inhibit T cell activation and proliferation

Recently, scientists have confirmed that cancer cells release exosomes carrying PD-L1, which spread directly from the tumor tissue to all parts of the body and comprehensively crack down on and suppress the human immune system. PD-L1 in exosomes has the same structure as it does on the surface of tumor cells and can also bind to PD-1 on T cells. Cell experiments showed that exosomes incorporated with PD-L1 can inhibit the proliferation of CD8+ T cells (Fig. 1). In mouse experiments, exosomes carrying PD-L1 promoted tumor growth and reduced the number of T cells in the spleen and lymph nodes [38]. Therefore, when the exosomes carrying PD-L1 reach the pre-metastatic microenvironment, the immune system is inhibited here, and the development of the pre-metastatic niche is promoted.

### Inhibitory immune cell recruitment

Tumor exosomes can cause recruitment of suppressive immune cells. After exosomes upregulate the expression of proinflammatory factors, the local inflammatory microenvironment induces tumor cells to produce chemokines and cytokines. These factors act synergistically with the exosomes produced by tumor cells to recruit tumor-associated macrophages (TAMs), tumor-associated neutrophils (TANs), regulatory T (Treg) cells and myeloid-derived suppressor cells (MDSCs) to distant secondary sites [5]. These immune cells may inhibit antitumor immune responses [39, 40]. Hematopoietic stem and progenitor cells can also differentiate into MDSCs, thereby promoting immunosuppression within the pre-metastatic niche [41].

### Impaired function of T cells, NK cells and antigen-presenting cells

Exosomes contain many substances that can impair immune cell function, thereby forming an immunosuppressive premetastatic microenvironment. Prolyl hydroxylase-mediated oxygen sensing can inhibit CD4 + and CD8 + T cell functions [42]. Cancer exosomes can cause NK cell dysfunction, inhibit antigen-presenting cells, block T cell activation, and enhance T cell apoptosis to block adaptive immune responses [43, 44]. In addition, four known immunomodulatory proteins have been identified in melanoma EVs, the combination of which reduces DC maturation in vitro [45].

### Molecular regulation

In addition to immunosuppression at the cellular level, some molecules are also involved in the immunomodulation of the pre-metastatic niche. During the process of MDSCs promoting immunosuppression in the pre-metastatic niche of liver cancer, TNF receptor-2 plays a supporting role [46]. In the pre-metastatic niche, ARG1 and reactive oxygen species (ROS) can inhibit antitumor T cells [47]. In addition to T cells, B cells are also inhibited. Through the TGF-βR1/TGF-βR2 signaling pathway, cancer-induced regulatory B (Breg) cells further potentiate the immunosuppression and metastasis function of MDSCs. Under the regulation of Breg cells, MDSCs produce more ROS and NO to inhibit CD8+ T cells [48].

### Immune surveillance of the heterogeneity of tumor-derived exosomes

It is well-known that tumor cells are heterogeneous, which can cause differences in tumor growth rate, tumor invasion, metastasis, drug sensitivity, and prognosis. Similarly, the latest research suggests that exosomes are also heterogeneous.

Unlike the above conclusions, “nonmetastatic” exosomes have the opposite effect of exosomes produced by metastatic tumor cells. Nonmetastatic exosomes can inhibit tumor cell metastasis by increasing the number of lung-patrolling monocytes, recruiting NK cells, and inducing macrophage differentiation and phagocytosis [49]. Moreover, the level of exosome PD-L1 in metastatic melanoma cells was significantly higher than that in primary melanoma cell exosomes [38]. This experiment confirmed the heterogeneity of exosomes derived from different cells.

To sum up, exosomes affect the immune response in pre-metastatic niches through multiple mechanisms. Exosomes are heterogeneous. In the pre-metastatic niche, the effects of exosomes on immunity were divided into positive and negative aspects. On the one hand, as positive effects (promoting immune surveillance), non-metastatic exosomes increase lung mononuclear cells, recruit NK cells and induce macrophage phagocytosis. On the other hand, as negative effects (causing immunosuppression), exosomes not only mediate immune escape through PD-L1, but also promote the recruitment of suppressive immune cells impairing immune cell function. In addition, some immunosuppressive molecules, such as TNF receptor-2, ARG1, ROS and NO, also inhibit the immune function of the pre-metastatic niche.

## Exosomes increase angiogenesis and vascular permeability in the pre-metastatic niche

The microenvironment prior to metastasis can increase angiogenesis and vascular permeability to facilitate metastasis. Studies have reported that exosomes released from hypoxic tumors are more likely to cause angiogenesis and vascular leakage [50, 51].

Mesenchymal-derived microvesicles activate endothelial cells via Akt phosphorylation and promote the formation of a metastatic vascular microenvironment [52]. Human kidney cancer stem cell-derived CD105-positive microvesicles can also stimulate angiogenesis, thereby promoting the formation of the pre-metastatic niche [53]. In addition, the exosome surface expresses soluble E-cadherin, which promotes tumor angiogenesis [54]. Furthermore, exosomes can express carbonic anhydrase 9 to promote angiogenesis [55]. In the pre-metastasis niche, new blood vessels not only transport TDSFs and EVs but also help bring circulating tumor cells to secondary sites. Then, exosomes begin to increase vascular permeability.

Peinado found that exosomes from the highly malignant B16-F10 enhanced lung endothelial permeability in mice compared to exosomes from nonmetastatic cell lines [19]. Melanoma-derived exosomes also induce vascular leakage and receptor tyrosine kinase Met to reprogram bone marrow progenitor cells in the niche [19]. It was observed by exosome tracing that 1833-BoT and 4175-LuT exosomes promoted pulmonary vascular leakage 24 h after injection [25]. MiR-105 secreted by metastatic breast cancer cells may disrupt the vascular endothelial barrier in the early metastatic niche, thereby increasing the vascular permeability of distant organs and promoting metastasis [56]. In addition, breast cancer cells can express matrix metalloproteinase (MMP) and cyclooxygenase. These enzymes promote vascular remodeling and increase vascular permeability to accelerate tumor cell metastasis [57].

A recent study showed that exosome secreted by colorectal cancer (CRC) cells was rich in miR-25-3p, which promoted angiogenesis and disrupted the tight junctions of vascular endothelial cells by targeting KLF2 and KLF4 [58]. In addition, the miR-25-3p of CRC cell-derived exosomes significantly induced vascular leakage and CRC metastasis in the liver and lung of mice [58]. Tumor-derived exosomes are considered to be the major drivers of the pre-metastatic niche. The above studies demonstrated that exosomes were involved in angiogenesis and increased vascular permeability in order to facilitate the formation of the pre-metastatic niche. Before the tumor is transferred, a microenvironment suitable for tumor metastasis has been created for tumor metastasis. This is another evidence that tumor-derived exosomes promote tumor metastasis.

## Tumor exosomes determine organotropism metastasis

If the tumor cells are regarded as seeds and the pre-metastasis niche is regarded as soil, then the exosomes are similar to fertilizers, which can make the barren land fertile and facilitate the colonization of tumor cells [59–63]. This hypothesis helps us understand exosome-mediated organ-specific metastasis.

**Fig. 2:**
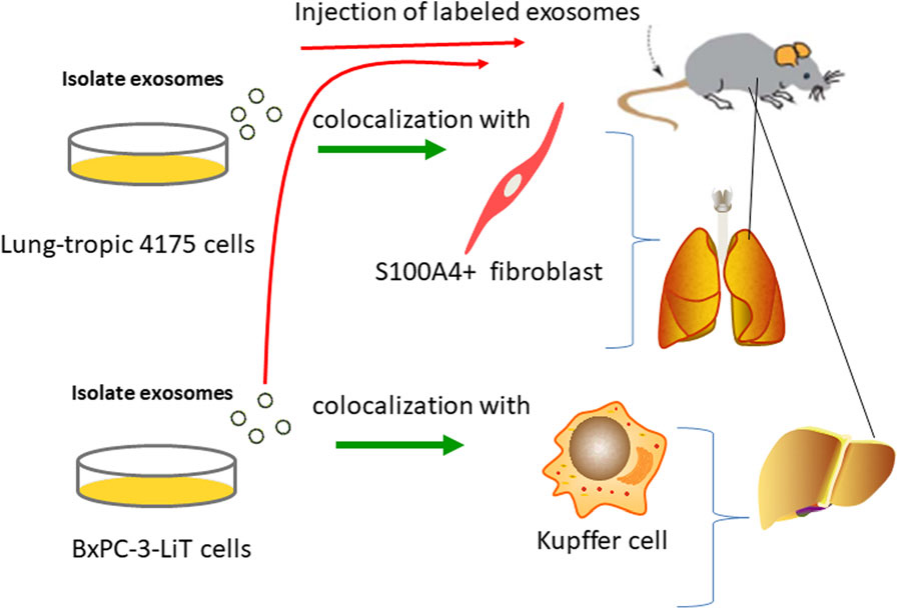
Different cells uptake tropic exosomes. Lung-tropic 4175 exosomes mainly colocalized with S100A4-positive fibroblasts in the lung; pancreatic cancer exosomes derived from BxPC-3-LiT cells mainly fused with Kupffer cells

Hoshino found that tumor-derived exosomes were important factors in organ-specific metastasis. Tumor-derived exosomes prepare a favorable microenvironment at future metastatic sites, thereby mediating nonrandom transfer patterns. First, future metastatic sites uptake exosomes. Second, exosomes redirect metastatic distribution. Third, ITGs on the surface of exosomes determine organotropism. Quantitative mass spectrometry and western blot analysis as well as qualitative mass spectrometry showed that ITGα6 was present in lung-directed exosomes, while ITGβ5 was present in liver-directed exosomes. These results indicate that the different expression of exosome ITGs is the basis of organotropism. Fourth, different cells absorb tropic exosomes. Lung-tropic 4175 exosomes mainly colocalized with S100A4-positive fibroblasts in the lung. By contrast, pancreatic cancer exosomes derived from BxPC-3-LiT cells mainly fused with Kupffer cells (Fig. 2). These data demonstrate that specific tissue-resident stromal cells differentially uptake tumor exosomes in metastatic target organs. Finally, exosome tropism requires ITGβ4 and ITGβ5. After knocking down ITGβ4 expression in 4175-LuT cells (4175β4KD), a more than threefold reduction in labeled ITGβ4KD exosomes in the lung was observed. Conversely, ITGβ4 overexpression in 1833-BoT exosomes was sufficient to increase exosome uptake in the lung. Similarly, ITGβ5 knockdown in BxPC-3-LiT exosomes decreased liver uptake by sevenfold compared with uptake by control BxPC-3-LiT exosomes. The above results demonstrate that exosome ITGs are responsible for organ-specific metastasis [25].

The above evidence proves that tumor cells prepare the receiving organs by releasing exosomes. Cancer cells spread from the origin to distant organs through blood circulation. The process of dissemination is not random, and cancer cells will preferentially look for specific organs under the guidance of exosomes and build nests there. This kind of destination-seeking behavior involves the interaction of “seeds” and “soil”. Seeds can fertilize the “soil” through exosomes before they reach the soil, consequently preparing for tumor metastasis.

## Stroma cell activation and remodeling of the extracellular matrix (ECM) in pre-metastatic niches

The matrix environment of the pre-metastatic niche is mainly composed of fibroblasts, endothelial cells and extracellular matrix (ECM). Fibroblasts not only produce inflammatory and growth factors but also express fibronectin (FN) and matrix metalloproteinase (MMP) [64]. Moreover, endothelial cells secrete proinflammatory factors S100A8 and S100A9 [65]. A prominent part of the pre-metastasis matrix of liver cancer is hepatic stellate cells, which can promote ECM deposition and upregulate the expression of VEGF, IL-1a and TGF-β [66, 67] . Inflammatory factors produced by stromal cells may contribute to the formation of local inflammatory microenvironments. Growth factors secreted by stromal cells may promote angiogenesis in the pre-metastatic niche. In addition, hypoxia is also an important factor of ECM. Hypoxia can induce the expression of HIF, VEGF and G-CSF, thereby promoting the formation of a pre-metastatic niche [68, 69].

Melanoma-derived exosomes can reprogram human adult dermal fibroblasts (HADF) to cause extracellular acidification [70]. Multiple types of BMDCs promote matrix remodeling in the pre-metastatic niche, and upregulation of fibronectin (FN) [2, 71]. Costa-Silva showed that exosome macrophage migration inhibitory factor (MIF) induces the release of TGF-β from Kupffer cells, which in turn promotes the production of FN in hepatic stellate cells (HSTCs). FN deposits subsequently promote the stagnation of bone marrow-derived macrophages and neutrophils in the liver, completing the formation of the pre-metastatic niche [72]. Exosomes expressing ITGα6β4 and ITGα6β1 co-localize with laminin, and exosomes expressing ITGαvβ5 colocalize with fibronectin [25]. Therefore, we hypothesize that specific exosome integrins may interact with extracellular matrices, and that deposited laminin and fibronectin may help increase the adhesion of extracellular matrices to facilitate colonization of circulating tumor cells.

## Roles of coding or non-coding RNA carried out by exosomes in the pre-metastatic niche

Tumor-derived exosomes have been reported to contain DNA fragments that provide exosome origin information. Once released into the peripheral cycle, the information contained in DNA fragments may direct the metastatic behavior of circulating tumor cells [73, 74]. Mutant genes were found in serum exosomes from patients with pancreatic cancer. For example, p53 and KRAS mutations have been verified [75]. In addition, DNA fragments with the ability to amplify the c-Myc gene were found in medulloblastoma cell-derived exosomes [76].

In addition, exosome proteins have been identified to be associated with the cell cycle, cell signaling process, cytoskeleton formation, apoptosis, oxidative stress, focal adhesions formation, and cell mobility [77]. Exosome integrins play an important role in the pre-metastatic niche, which has been described in detail above. Studies have shown that osteopontin derived from tumor microbubbles contributes to the colonization of tumor cells and BMDC mobilization [78].

Furthermore, microRNAs associated with metastasis in exosomes have also been reported [79]. Tumor-secreted microRNAs bind to TLR8 in human immune cells, triggering tumor metastasis [80]. Brain astrocyte-derived exosomes mediate PTEN targeting of miRNA-19a, thereby promoting PTEN silencing in tumor cells. Decreased PTEN upregulates the expression of chemokine CCL2, which recruits bone marrow cells in the brain [81]. Exosomes derived from prostate cancer contain differentially expressed genes (DEGs), such as miR-21-5p and miR-139-5p. These microRNAs derived from exosomes cooperate to adjust the pre-metastatic niche [82]. Studies have reported that lncRNAs have important functions for pre-metastatic niches [83]. Multiple studies have shown that lncRNAs can be used as biomarkers for tumor diagnosis [84–86] . Circular RNA (circRNA) is a closed loop structure, and its expression is more stable and less prone to degradation. Circular RNA has spatiotemporal specificity and is highly conserved among species. Recently, the role of circRNA in exosomes has been reported. In the future, the function of circRNA in the pre-metastatic niche may be gradually excavated.

From the above studies, we conclude that both coding and non-coding RNA derived from exosomes promote the formation of pre-metastatic niches. At the present, the induction of pre-metastatic niche formation by exosome miRNAs has attracted wide attention. In the future, exosome-derived lncRNA and circRNA will also show their prominence.

## Molecules carried by exosomes may be tumor biomarkers

In recent years, research on finding biomarkers for detecting diseases has grown exponentially. Some studies have used exosomes containing non-coding RNA as potential biomarkers for detecting different malignancies. MiRNAs isolated from tumor-derived exosomes are more stable and therefore considered to be more reliable biomarkers. In addition, exosome-derived lncRNA and circRNA are also fascinating potential biomarkers.

### Biomarkers for tumor diagnosis

The levels of miR-21, miR-150, miR-223, and miR-1229 in serum exosomes of colorectal cancer (CRC) patients were significantly higher compared to those of the control group. Similarly, in vitro experiments revealed that these exosome miRNAs were expressed at higher levels in CRC cell lines than in nontumor cell lines [87]. In gastric cancer (GC), plasma LINC00152 levels in patients with gastric cancer were significantly higher than in healthy controls [88]. The above findings suggest that molecules carried by exosomes can be potential biomarkers for the early detection of disease.

### Biomarkers for metastasis predication

Scientists have found that exosomes in the blood of melanoma patients, especially those with metastatic melanoma, have significantly higher PD-L1 levels than those of healthy individuals [38]. Hoshino found more highly-expressed ITGβ4 in exosomes from patients with lung metastasis compared with those from in situ cancer or liver metastasis patients. The exosomes isolated before lung metastasis of breast cancer patients express the highest level of ITGβ4. In addition, levels of ITGαv in the exosomes of patients with liver metastases were elevated compared with those of patients with orthotopic tumors or lung metastases [25]. In gastric cancer patients, serum exosomes ZFAS1 are highly expressed. High levels of ZFAS1 were significantly associated with lymphatic metastasis and TNM staging [89]. Moreover, low levels of miR-217 in serum exosomes are associated with lymph node metastasis or distant metastasis [90].

### Biomarkers for the evaluation of prognosis

For patients with prostate cancer, upregulation of miR-375 and miR-1290 may indicate a lower overall survival rate [91]. It has been reported that elevated serum exosome CRNDE-h is closely related to poor prognosis in patients with CRC [92]. In addition, there is a significant difference in the expression of miR-17-92 clusters in CRC patients with poor prognosis compared with healthy patients [93]. PD-1 levels are associated with the prognosis of classical Hodgkin’s lymphoma. Inhibiting the expression of PD-1 can improve the prognoses of patients [94]. High expression of PD-L1 in gastric cancer tissues may be associated with poor prognosis [95]. In patients with triple-negative breast cancer, PD-L1 is highly expressed in the tumor tissues [96].

In short, exosome-derived PDL-1 and noncoding RNA affect tumor biological functions such as invasion, metastasis and proliferation. Therefore, these molecules have the potential to affect prognosis.

### Biomarkers for the evaluation of treatment effectiveness

Recently, in 44 melanoma patients treated with Keytruda, scientists found that among the 21 patients who responded to treatment, the level of exosome PD-L1 in the blood before treatment was significantly lower than the level of exosome PD-L1 of the other 23 patients who did not respond to treatment. In terms of prognosis, patients with low levels of PD-L1 in blood exosomes had a better prognosis. In the future, monitoring PD-L1 or other biomarkers on circulating exosomes may be an effective way to predict and observe therapeutic effects [38].

Traditional tissue biopsy is invasive and cannot be used for the early diagnosis of tumors. For years, researchers have been looking for non-invasive cancer detection tools. Currently, with the in-depth study of plasma exosomes, liquid biopsy has been considered for the early diagnosis of tumors [97]. Exosomes secreted by tumor cells carry different types of contents, and these molecules have multiple key biological activities. [98]. Exosomes can be abundantly released from cancer cells and can be broadly distributed in body fluids. Exosomes are very stable and are capable of protecting proteins and nucleic acids with its lipid bilayer. In short, exosomes possess obvious advantages in terms of quantity, stability, and accessibility.

Previous studies have shown the advantages of exosomes in predicting metastasis, prognosis and therapeutic effects on tumors. However, the credibility of a single molecule is limited, and some researchers suggest using a combination of the most reliable biomarkers to improve the sensitivity and specificity of detecting tumors [99].

## Exosome-mediated potential anti-tumor therapy

### Exosome-mediated chemotherapy resistance

Currently, chemotherapy is still one of the main methods of cancer treatment. However, tumor cell resistance to chemotherapy makes treatment difficult. Therefore, understanding the mechanisms of chemical resistance and identifying key molecules are the basis for solving the current dilemma.

Some studies have linked exosomes to chemotherapy resistance by the action of cargo miRNAs on recipient cells. It has been observed that miRNAs are tumor suppressors, leading to uncontrolled cell growth and resistance to anti-tumor drugs [100]. Furthermore, the chemical resistance of 5-FU can result in the enhanced invasion and migration of tumor cells. This phenomenon is related to the loss of miR-200 [101]. P73 is an isoform of p53. Both in vivo and in vitro experiments have shown that ΔNP73 can increase the proliferation of CRC cells and confer resistance to oxaliplatin by exosome transfer [102].

### The pre-metastatic niche provides new ideas for targeted therapy

Targeted therapy is the treatment of established carcinogenic sites at the cellular and molecular levels. When a targeted drug enters the body, it specifically binds to the oncogenic site, causing the tumor cells to die without affecting normal tissue cells, so molecular targeted therapy agents have also called “bio-missiles”. With the development of molecular biology, molecular targeted therapy has gradually become a new means of cancer treatment in recent years.

Although targeted therapy has been recognized by the medical community for its unique advantages, the effects on cancer treatment are limited. There are significant individual differences in targeted therapeutic effects. Drugs that are useful to some individuals may not be effective for other patients. Therefore, finding a powerful target in the early process of tumor development is the key to breaking through the bottleneck of targeted therapy.

Tumor-derived exosomes transmit information between cells to promote the formation of pre-metastatic niches. Thus, blocking the delivery of exosome cargos to recipient cells may be a powerful strategy for preventing tumor metastasis. There are many targets that could be used to inhibit the formation of pre-metastatic niches (for instance, blocking the production of proinflammatory factors, inhibiting the recruitment of BMDCs, preventing angiogenesis and vascular penetration, destroying the local matrix, and reactivating the anti-tumor immune response). These targets could develop potential ways to prevent and control cancer metastasis in the future.

## Conclusions

**Fig. 3:**
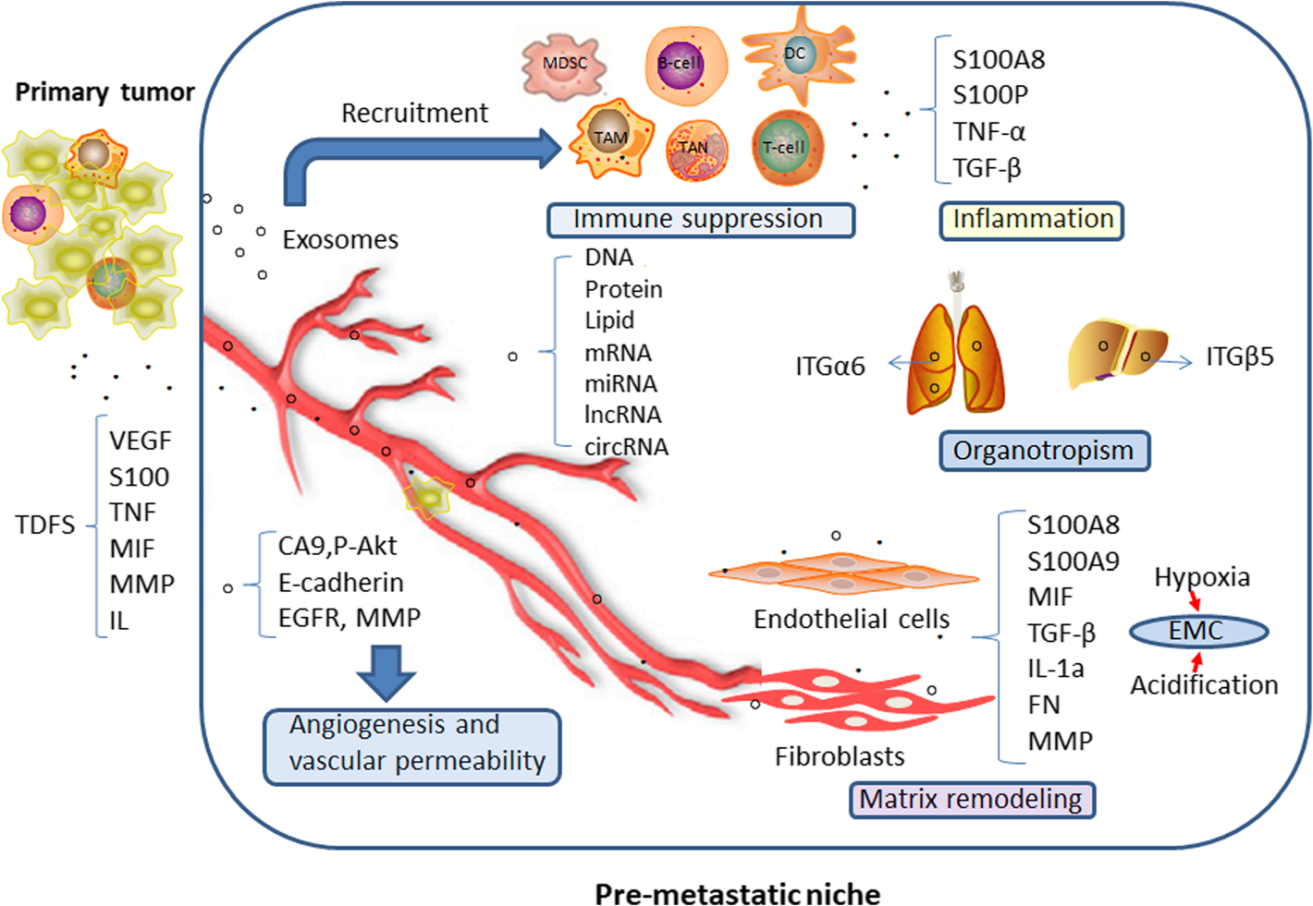
Effects of exosomes on tumor pre-metastatic niche formation. Exosomes promote the upregulation of inflammatory molecules; exosomes induce immune suppression; exosome increase angiogenesis and vascular permeability; exosome determine organotropism metastasis; Exosome promote matrix remodeling

In summary, exosomes play multiple roles in the development of pre-metastatic niches. Exosomes can not only promote the upregulation of inflammatory molecules, induce angiogenesis and infiltration, and regulate immune function in both directions but also determine organotropism metastasis and cause extracellular matrix remodeling [103] (Fig. 3).

Many studies have confirmed that exosomes can promote the formation of pre-metastatic niches. However, the functions, mechanisms, and contribution rates of exosomes remain to be further explored. The problems that need to be solved urgently include 1. What is the difference in effect between tumor-derived exosomes and immune cell- or stromal cell-derived exosomes on the pre-metastatic niche? 2. The composition of exosomes is complex, so what role do these components play? Is there an interaction between the different components? 3. After the primary tumor is surgically removed, does the effect of the exosomes on the pre-metastatic niche terminate? 4. What is the reliability and validity of using exosomes as biomarkers to judge tumor metastasis? 5. How can the theoretical basis of exosomes and pre-metastatic niches be applied to clinical treatment?

To conclude, exosomes play an essential role in the pre-metastatic niche, and further exploration of the function of exosomes in the pre-metastatic niche will help to determine treatments in the early stage of cancer. In addition, circulating exosomes, as biomarkers, have great potential in liquid diagnostics. As exosomes have the biological role of transporting cargo, exosomes may be used as a carrier for targeted drugs to accurately destroy tumor cells [104]. In the future, tumors are expected to become as chronic as diseases such as diabetes. Just as people with diabetes monitor blood glucose with a blood glucose meter, tumor patients will be able to track tumor progression through exosome biomarkers.

## Abbreviations

APCs: Antigen presenting cells
BMDCs: Bone marrow-derived cells
CRC: Colorectal cancer
DC: Dendritic cell
ECM: Extracellular matrix
EVs: Extracellular vesicle
FN: Fibronectin
GC: Gastric cancer
HADF: Human adult dermal fibroblasts
HSTCs: Hepatic stellate cells
KCs: Kupffer cells
LncRNA: Long noncoding RNA
MIF: Macrophage migration inhibitory factor
NK: Natural killer cells
PD-1: Programmed death receptor 1
PD-L1: Programmed death ligand 1
TAMs: Tumor-associated macrophage
TANs: Tumor-associated neutrophil
TDSFs: Tumor-derived secreted factors
VEGF: Vascular endothelial growth factor
